# PR11‐364P22.2/ATF3 protein interaction mediates IL‐1β‐induced catabolic effects in cartilage tissue and chondrocytes

**DOI:** 10.1111/jcmm.16561

**Published:** 2021-05-26

**Authors:** Xilei Li, Yusheng Li, Xucheng Yang, Runzhi Liao, Liang Chen, Qulian Guo, Junxiao Yang

**Affiliations:** ^1^ Department of Anesthesiology Xiangya Hospital Central South University Changsha China; ^2^ National Clinical Research Center for Geriatric Disorders Xiangya Hospital Central South University Changsha China; ^3^ Department of Orthopaedics Xiangya Hospital Central South University Changsha China

**Keywords:** activating transcription factor 3, cartilage, chondrocytes, IL‐1β, long non‐coding RNA, osteoarthritis, RP11‐364P22.2

## Abstract

Osteoarthritis (OA) is a degenerative joint disease which lacks effective medical treatment due to ill‐defined molecular mechanisms underlying the pathology. Inflammation is a key factor that induces and aggravates OA. Therefore, the current study aims to explore roles of the dysregulated long non‐coding RNAs in the pro‐inflammatory cytokine IL‐1β‐mediated catabolic effects in cartilage tissue and chondrocytes. We identified RP11‐364P22.2 as dysregulated in OA patient‐derived cartilage tissues and highly responsive to IL‐1β stimulus. RNA pull‐down coupled with mass spectrometry demonstrated that RP11‐364P22.2 physically binds to activating transcription factor 3 (ATF3) and thus increases the protein stability and facilitates its nuclear translocation. Loss‐ and gain‐of‐function assays indicated that the interaction between RP11‐364P22.2 and ATF3 is indispensable for the detrimental effects of IL‐1β including growth inhibition, apoptosis induction as well as degradation of the key chondrocyte structural proteins of type II collage and Aggrecan and synthesis of the extracellular matrix‐degrading enzyme MMP13 in chondrocytes. In vivo, depletion of the RP11‐364P22.2 effector ATF3 drastically prevented OA development in the rats with surgical destabilization of the medial meniscus (DMM). These results highlight the important roles of lncRNAs in the pathogenesis of OA and indicate the RP11‐364P22.2/ATF3 regulatory axis as a potential therapeutic target of inflammation‐induced OA.

## 1 INTRODUCTION

Osteoarthritis (OA), also called degenerative joint disease, is the most prevalent form of arthritis. Clinical symptoms of OA include joint pain in hands, neck, lower back, knees or hips, degeneration of articular cartilage, limited intra‐articular inflammatory synovitis and changes in the surrounding and subchondral bones of the joints.^1^ Aetiologies of OA are diverse and complicated.^2^, ^3^, ^4^, ^5^ Due to the poorly understood molecular mechanisms underlying OA initiation and development, there is no effective intervention curing degraded cartilage or restraining disease progression.

It has been long aware that early OA development is accompanied with chronic low‐grade inflammation. Unlike that in rheumatoid arthritis, synovial fluid white blood cell count in OA barely exceeds 1000‐2000/mL.^6^ At the molecular level, the inflammation in OA joints is characterized by the presence of massive amounts of pro‐inflammatory mediators including cytokines such as TNF, IL‐1β and IL‐6 and chemokines which are involved in the innate immune response to joint damage.^7^

Long non‐coding RNA (lncRNA) is a superfamily of transcribed RNA molecules which are more than 200 nucleotides (nt) in length and do not encode proteins.^8^ LncRNA is pathogenic to many human diseases including cancer, Alzheimer's disease and diabetes.^9^ Increasing evidence shows that lncRNAs are also key regulators of cartilage formation, bone and cartilage homeostasis, and thus affect the progress of OA.^10^ However, the roles of lncRNAs in OA development, especially the inflammation process, remain largely unknown.

In the current study, we demonstrated that RP11‐364P22.2 is not only over‐expressed in OA cartilage tissues and chondrocytes, but also highly responsive to the stimulus of the pro‐inflammatory cytokine IL‐1β. Binding of RP11‐364P22.2 to activating transcription factor 3 (ATF3) stabilizes the protein and facilitates its nuclear translocation, which is indispensable for OA development in the rats underwent surgical destabilization of the medial meniscus (DMM) in vivo. In vitro mechanism studies displayed that the interaction between RP11‐364P22.2 and ATF3 is essential for IL‐1β‐induced growth inhibitory and apoptotic effects as well as degradation of the structural proteins and synthesis of the extracellular matrix (ECM)‐degrading enzyme MMP‐13 in chondrocytes. These results highlight the importance of lncRNAs in the pathogenesis of OA and indicate the RP11‐364P22.2/ATF3 regulatory axis as a potential therapeutic target of inflammation‐induced OA.

## 2 MATERIALS AND METHODS

### 2.1 Patient samples

Osteoarthritis articular cartilage tissues were collected from OA patients (n = 10) undergoing the entire knee replacement surgery, while the amputations from non‐OA and rheumatoid arthritis patients (n = 10) were used as controls. All the patients were informed of their participation in the current study and gave their informed consent. This study was approved by the Ethics Committee of the Ethics Committee of Xiangya Hospital (Changsha, China) and performed in accordance with the Declaration of Helsinki.

### 2.2 Cell line and reagents

Human chondrosarcoma cell line SW1353 was purchased from Chinese Academy of Sciences Cell Bank (Shanghai, China) and cultured in Dulbecco's Modified Eagle Medium (DMEM)/F12 containing 10% foetal bovine serum (FBS) in humidified atmosphere 5% CO2 in air at 37°C. IL‐1β was obtained from Roche Diagnostics (Germany), and cycloheximide (CHX) was purchased from Sigma‐Aldrich.

### 2.3 Gene expression

For lncRNAs and mRNA expression studies, total RNA was extracted using TRIzol reagent (Invitrogen) followed by reversed transcription using RevertAid^TM^ H Minus First Strand cDNA Synthesis Kit (Fermentas). Quantitative real‐time PCR was conducted using SYBR Green PCR Master Mix (ABI 4309155) in ABI7900 PCR machine. The sequences of primers are shown in Table 1.

**TABLE 1:** Sequence information

Gene	Forward (5'‐3')	Reverse (5'‐3')
qPCR primer sequences		
RP11‐364P22.2	GCTCCCTAAGCCCAACTTTT	GAGGCCAAGGCTAGAGGATT
RP11‐432M8.8	ATGAAGCGTCAGCCTCAACT	CGACACACTTCGTAGCGAGA
RP11‐399K21.10	CTAGCCTCGGGGACCTCA	TAGCTGCAGCAAGAGTGGAA
AC019109.1	GGCCTGTGGGTACCACTCTA	CCATGTGTTTTCTGCCAATG
ATF3	CAAGTGCATCTTTGCCTCAA	CCACCCGAGGTACAGACACT
β‐actin	ACCCTGAAGTACCCCATCGAG	AGCACAGCCTGGATAGCAAC
Primers U1	GGGAGATACCATGATCACGAAGGT	CCACAAATTATGCAGTCGAGTTTCCC

siRNA	Sense(5'‐3')	Antisense(5'‐3')
siRNA sequences		
siRNA‐RP11‐364P22.2‐381	UUCUAUUUAUAUCUUAUUGTT	CAAUAAGAUAUAAAUAGAATT
siRNA‐RP11‐364P22.2‐503	UAUUUGAGACCAGGAUUUCTT	GAAAUCCUGGUCUCAAAUATT
siRNA‐RP11‐364P22.2‐666	AUACAUUGCAUGAGAAUACTT	GUAUUCUCAUGCAAUGUAUTT
Negative control FAM	UUCUCCGAACGUGUCACGUTT	ACGUGACACGUUCGGAGAATT

The oligonucleotide sequences for RNA pull‐down	
RP11‐364P22.2‐sense‐F	TAATACGACTCACTATAGGGAGACGGGGTGGCGGCCGGGCAGAGGCT
RP11‐364P22.2‐sense‐R	TCTTTCTACTGGAGATACCAACTAC
RP11‐364P22.2‐antisense‐F	CGGGGTGGCGGCCGGGCAGAGGCT
RP11‐364P22.2‐antisense‐R	TAATACGACTCACTATAGGGAGA TCTTTCTACTGGAGATACCAACTAC
Antisense oligo probe sequences	
AS‐oligo1(37‐87bp)	5'‐CCCCGCGGGGCCCGAGGGCAAGGAGCAGCCGCCTGCCTTGGCCTCCCAAAG‐3'
AS‐oligo2(88‐138bp)	5'‐CGCCGCGCCGGCGAGCGCCGCCCGGGAGGCAGCGGCTGGAGGAGCGGACGG‐3'
AS‐oligo3(162‐227bp)	5'‐GGAGCCGCTGCCGCGACCGACCGCAGCCGCCGCCGCCCGACCGCCGGGAGGATGGAGTTCAGCGGG‐3'
Bio‐control oligo	5' TCCTTCCTCTCTTTCTCTCCCTTGTGA 3'

### 2.4 Primary human chondrocytes culture

Preparation of primary human articular chondrocytes was performed as described by Manning and Bonner.^11^ Briefly, the disinfected cartilage was first cropped into small fragments. Chondrocytes were then released by sequential digestion with 0.25% trypsin (Gibco) and 1 mg/mL type II collagenase (Sigma) at 37°C and cultured with DMEM/F12 containing 10% foetal bovine serum (FBS) in a 37°C water‐jacketed CO_2_ incubator. The primary chondrocytes exceeding five passages were abandoned due to an irrepressible dedifferentiation caused by repetitive subculture.

### 2.5 Immunofluorescence

The monolayer chondrocytes on the glass cover slide were fixed with 4% paraformaldehyde and blocked with 5% normal goat serum. After an overnight incubation with primary antibodies (Table 2) at 4°C, cells were washed three times with PBS and incubated with Alexa Fluor‐conjugated secondary antibody for 1 hour at room temperature. DAPI was used for nuclear counterstain.

**TABLE 2:** Antibody information

Primary antibodies	MW (kD)	Dilution	Company/Catalogue	Secondary antibodies	Dilution
MMP‐13	≈54	1:1000	Ptgcn,18165‐1‐AP	Goat anti‐rabbit IgG/HRP	1:4000
Type II collagen	≈65	1:500	Ptgcn,27102‐1‐AP	Goat anti‐rabbit IgG/HRP	1:4000
Aggrecan	≈150	1:500	Abcam,ab3778	Goat anti‐mouse IgG/HRP	1:4000
Cleaved caspase‐3	≈17	1:500	Abcam,ab2302	Goat anti‐rabbit IgG/HRP	1:4000
NFKB‐p65	60	1:500	SAB,21210	Goat anti‐rabbit IgG/HRP	1:4000
p‐NFKB‐p65 (Ser536)	60	1:500	SAB11014	Goat anti‐rabbit IgG/HRP	1:4000
ATF3	≈29	1:500	Novusbio,NBP1‐85816	Goat anti‐rabbit IgG/HRP	1:4000
β‐actin	42	1:2000	Ptgcn,66009‐1‐Ig	Goat anti‐mouse IgG/HRP	1:4000
Histone H3	≈17	1:1000	CST,#4499	Goat anti‐rabbit IgG/HRP	1:4000

### 2.6 Cell proliferation

The time‐dependent chondrocytes proliferation was assessed using MTT Cell Proliferation and Cytotoxicity Detection Kit (KeyGEN Biotech, #KGA312). Briefly, cells were seeded at a density of 1 × 10^4^ cells per well in 96‐well plates and treated with IL‐1β for the indicated time. By the end of the treatment, an equal volume of MTT reagent was added into each well, and the plate was incubated at 37°C for 3 hours. Carefully remove the supernatant without disturbing the attached cells, add 150 µL MTT solvent into each well followed by shaking on an orbital shaker for at least 15 minutes in the dark and read the absorbance at OD = 590 nm.

### 2.7 Cell apoptosis

Apoptosis and cell death in chondrocytes were examined through flow cytometry using Annexin V‐PE/7AAD apoptosis kit (Abnova, #KA3809) based on the instruction of the manual. Briefly, the treated chondrocytes were washed twice with cold staining buffer and then resuspended in Annexin V Binding Buffer. Add sequentially PE Annexin V and 7AAD viability staining solution, and incubate for 15 minutes at room temperature in the dark. Add Annexin V Binding Buffer up to 500 µL of total volume in each tube and analyse by flow cytometry.

### 2.8 Western blot

Total protein was extracted using RIPA buffer supplemented with protease inhibitor cocktail (Sigma #P8340) from each experimental group of chondrocytes. 30 µg of whole cell extracts was fractionated by SDS‐PAGE and transferred to a nitrocellulose membrane. After blocking with 5% non‐fat milk for 1 hour at room temperature, the membrane was incubated with the primary antibodies (see Table 2) at 4°C overnight. Membranes were then washed three times with TBST (10 mmol L^−1^ Tris, pH 8.0, 150 mmol L^−1^ NaCl, 0.5% Tween 20) for 15 minutes at room temperature and incubated with a 1:4000 dilution of horseradish peroxidase‐conjugated secondary antibody for 1 hour. Blots were washed three times with PBST and developed with the ECL reagent (Pierce). Cytoplasmic and nuclear fractions of the chondrocytes were prepared according to the instructions of the nuclear/cytoplasmic isolation kit (Thermo Fisher Scientific, USA).

### 2.9 Fluorescence in situ hybridization (FISH)

The monolayer chondrocytes grown on glass slides were fixed by 4% paraformaldehyde and permeabilized with 70% ethanol overnight at 4°C. Cells were then washed with 10% formamide in 2× sodium citrate buffer (SSC, 3 mol L^−1^ NaCl, 0.3 mol L^−1^ sodium citrate, pH 7) and subjected to hybridization using oligonucleotide‐modified probes for human lncRNA RP11‐364P22.2 and a scramble control in 10% formamide, 2× SSC and 10% dextran sulphate (w/v) overnight at 37°C in a humidified chamber. After washing with 10% formamide in 2× SSC, the fluorescence was observed and imaged using a confocal microscope.

### 2.10 RNA pull‐down assay

The full‐length sense and antisense RP11‐364P22.2 were amplified using primers listed in Table 1 and labelled with biotin by Biotin RNA Labeling Mix (Roche) and the Riboprobe Systems with T7 RNA polymerase (Promega). For RNA pull‐down assay, cell lysates from ~2.0 × 10^7^ chondrocytes were pre‐incubated with 3 µg of the purified biotin‐labelled RNA probes for 4 hours at room temperature followed by adding streptavidin magnetic beads (Thermo, USA) and inverted overnight at 4°C. The proteins were then fractionated by SDS‐PAGE electrophoresis. After proper fixation, the gel was treated with protein treatment solution (20% ethanol, 5% acetic acid, 75% water and 4 mg dithiothreitol) and rinsed sequentially with 0.5% dichromate and water. Silver staining was performed by equilibrating the gel with 0.1% silver nitrate for 30 minutes followed by incubation in complex formation solution 0.02% paraformaldehyde, 3% sodium carbonate (pH12). The complex formation was stopped by addition of 1% acetic acid. The unique protein bands pulled down by sense but not the antisense RP11‐364P22.2 were further analysed by mass spectrometry and retrieved in human proteomic library. For assessing the ATF3 binding sites in RP11‐364P22.2, cell lysates were pre‐incubated with synthesized unlabelled antisense oligo targeting specific regions of the lncRNA (Table 1) at room temperature for 1 hour followed by adding sense biotin‐labelled RP11‐364P22.2 full‐length RNA probes and proceeded with the standard pull‐down procedure. The bound proteins in the pull‐down product were analysed by Western blotting using ATF3 antibody (Table 2).

### 2.11 RNA immunoprecipitation (RIP)

RNA immunoprecipitation was performed using Magna RIP^TM^ RNA‐binding protein immunoprecipitation kit (Millipore, MA) according to the manual instruction. Briefly, ~2.0 × 10^7^ cells were lysed in 100 µL RIP lysis buffer containing protease inhibitor cocktail and RNase inhibitor. For each immunoprecipitation, 50 µL of magnetic beads was pre‐washed by RIP wash buffer on a magnetic separator and resuspended in 100 µL of RIP wash buffer containing ~5 µg of ChIP grade anti‐ATF3 antibody (Abcam, #ab254268). The beads/antibody complex was incubated with rotation for 30 minutes at room temperature followed by washing with RIP wash buffer twice on the magnetic separator to remove the unbound antibodies and resuspended in 0.5 mL RIP wash buffer. The cell lysate was then added into RIP immunoprecipitation buffer containing the prepared beads/antibody complex and incubated with rotation overnight at 4°C. The normal human IgG antibody was used as a negative control in the assay. Next day, the supernatant was removed on the magnetic separator and the remaining beads were washed with cold RIP wash buffer for 6 times to remove the unbound RNAs. The resultant immunoprecipitate was further incubated with proteinase at 55°C for 30 minutes to digest the proteins. The supernatant containing the immunoprecipitated RNAs was separated from the beads on the magnetic separator and purified thereafter through phenol:chloroform:isoamyl alcohol extraction.

### 2.12 RNA interference

Sequences of small interfering RNA (siRNA) oligonucleotides targeting RP11‐364P22.2 and the negative control siRNA are listed in Table 1. For in vitro RNA interference, chondrocytes were transfected with 100 nmol L^−1^ of either the targeting or control siRNA using Lipofectamine^TM^ 2000 (Invitrogen; Thermo Fisher Scientific, Inc.) for 48 hours and subject to the functional assays. Lentiviral construct expressing rat ATF3 shRNA as well as the empty lentiviral vector were purchased from GeneChem (Shanghai, China) for depletion of *ATF3* in rat knee cartilage tissue in vivo.

### 2.13 Bioinformatics

Data for LncRNA expression Venn diagram were retrieved from three independent human OA patient‐derived data sets. Among these studies, Fu et al (2015) characterized lncRNAs expression profile in human osteoarthritic cartilage using microarray,^12^ while Li et al (2019) performed whole transcriptome sequencing assessing the expression profiles of mRNAs, lncRNAs and circRNAs in human OA cartilage tissue.^13^ In the study by Pearson et al (2016), the authors investigated the lncRNA expression in primary human hip OA chondrocytes following IL‐1β stimulation.^14^ The common signature lncRNAs in the current study were obtained by intersecting the upregulated and IL‐1β‐induced lncRNAs (*P* ≤ 0.05, log2 fold change ≥2) in these studies. For assessing RP11‐36P22.2 expression, the expression data were retrieved, respectively, from data set in the study by Li et al (2019) ^13^ as well as the data set of GSE117999 in the study by Brophy et al (2018), in which transcriptome comparison of cartilage from human subjects with and without osteoarthritis was conducted by microarray.^15^ For comparing ATF3 expression between normal and OA cartilage tissues, the expression data were retrieved, respectively, from the data sets of GSE117999 and GSE51588 in the study by Chou et al (2013), in which microarray was applied to assess gene expression profiling in human OA subchondral bone.^16^ In silico prediction of RP11‐364P22.2/ATF3 interaction was conducted using catRAPID (http://s.tartaglialab.com/page/catrapid_group).

### 2.14 Rat model of OA

Animal experiments were approved by the Ethics Committee of Xiangya Hospital (Changsha, China). Male SD rats (10‐week‐old, n = 12) were randomly divided into three groups. Before the surgery, rats were anaesthetized with isoflurane and the right knee area and lower leg were prepared for surgery. For the surgical DMM, a skin incision was made from the distal patella to the proximal tibial plateau. The join capsule immediately medial to the patellar tendon was incised and spread open with scissors. The patellar tendon was retracted, and the cranial meniscotibial ligament of the medial meniscus (MMTL) was exposed by a blunt dissection of fat pad. The MMTL was sectioned using a micro‐surgical knife, and then the wound was sutured. For in vivo ATF3 knock‐down, lentivirus (1 × 10^9^ PFU, 20 µL) expressing ATF3 shRNA was injected into the knee joints of recipient rats 1 week after the surgery (20 µL per joint per rat twice per week for 4 weeks). The experimental rats were killed, and knee joints were harvested by 8 weeks after the surgery.

### 2.15 Safranin‐O staining

Knee joints were harvested and fixed using zinc‐buffered formalin (Z‐Fix; Anatech, Battle Creek, MI) followed by decalcification in TBD‐2 (Thermo Fisher) for 12 hours on a shaker. The decalcified tissues were then embedded in paraffin. Knee joint sagittal sections were cut at 4 µm thick from the medial compartment at the junction between the middle region of the menisci and the anterior/posterior horns. The sections were then subject to staining with Safranin‐O/Fast Green. The histological assessment was conducted as suggested by the OARSI scoring system.^17^

### 2.16 Statistical analysis

Data are expressed as mean ± standard deviation of three independent experiments. Two‐tailed Student's *t* test was performed for determining a difference between two groups, while one‐way ANOVA analysis followed by Tukey's honestly significant difference post hoc test was conducted for comparing two‐group difference among the multiple groups using GraphPad Prism 8 software. For MTT assay results, only treatment or genotype effect was considered by comparing each experimental group mean with the control group mean within each time point using two‐way ANOVA analysis followed by Dunnett's multiple comparisons test. *P < 0.05, **P < 0.01, ***P < 0.001, n.s., non‐significant.

## 3 RESULTS

### 3.1 RP11364P22.2 is highly expressed in OA cartilage tissues and responsive to IL‐1β

**FIGURE 1:**
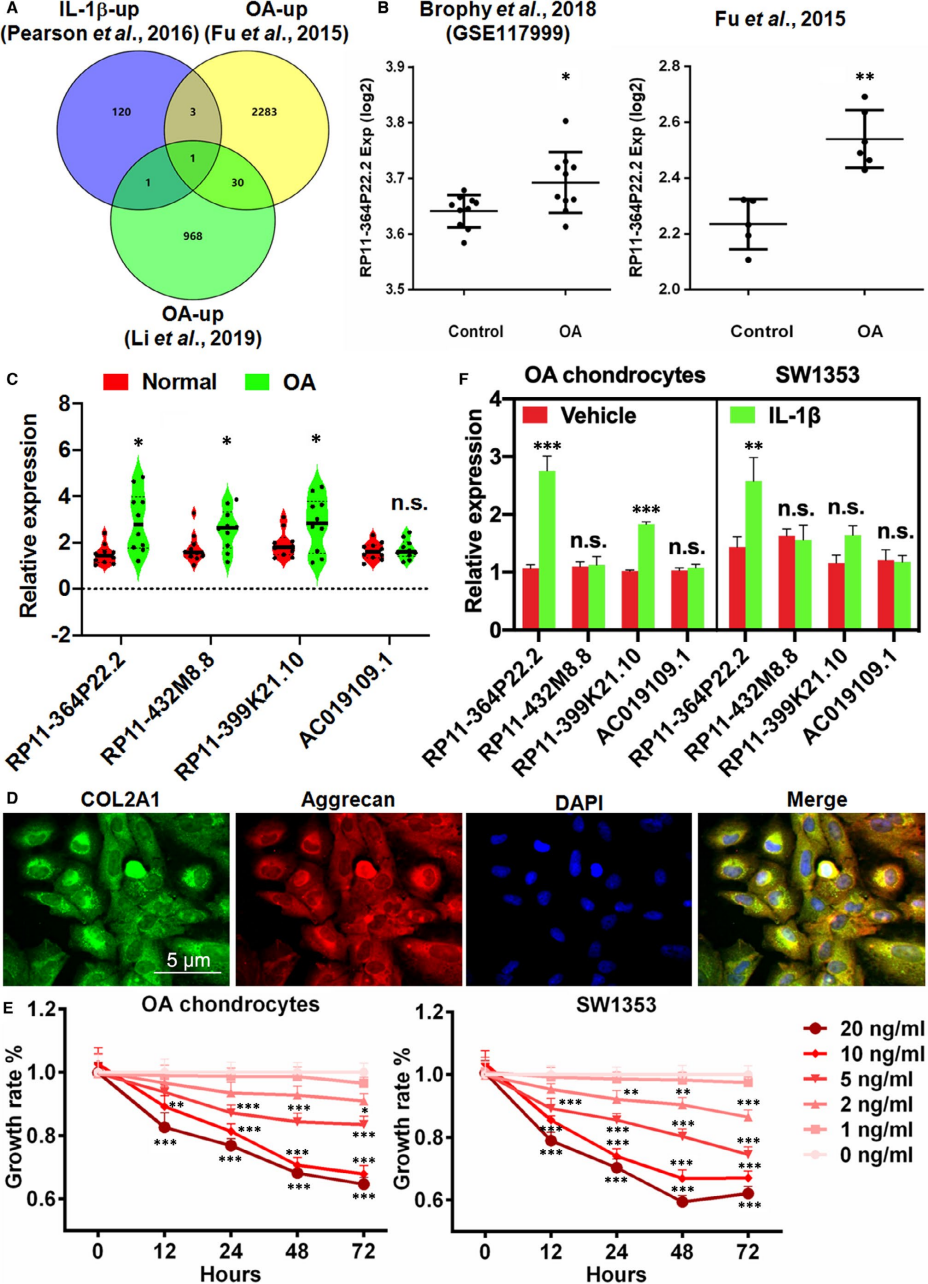
IL‐1β induces RP11‐364P22.2 expression in OA cartilage tissue and chondrocytes. A, Venn diagram showing the upregulated IL‐1β‐responsive lncRNAs in three independent OA patients‐derived data sets. B, Comparison of RP11‐364P22.2 expression in normal (Control) and OA cartilage tissues. C, qPCR analysis of expression of the candidate lncRNAs in normal and OA patient‐derived cartilage tissues. D, Immunofluorescence showing expression of type II collagen and Aggrecan in primary chondrocytes. DAPI was used as nuclei counterstain. E, MTT assay showing dose‐ and time‐dependent growth inhibitory effects of IL‐1β on the chondrocytes. F, qPCR analysis comparing expression of the candidate lncRNAs in primary chondrocytes and SW1353 in the absence or presence of IL‐1β

Osteoarthritis is accompanied with a simultaneous inflammation from early stage, and IL‐1β is one of the key inflammatory cytokines involved in the pathogenesis of the disease.^18^ To explore those essential lncRNAs involved in IL‐1β‐induced OA, we retrieved and interrogated lncRNAs that are not only upregulated in OA cartilage tissues but also highly responsive to IL‐1β exposure from three independent previous studies.^12^, ^13^, ^14^ Interestingly, RP11‐364P22.2 turned out as the unique signature in the intersection of all three databases (Figure 1A and B). We confirmed by qPCR the expression of RP11‐364P22.2 and the other three lncRNAs including RP11‐432M8.8, RP11‐399K21.10 and AC019109.1 which were only partially overlapped between data sets in the studies by Pearson et al (2016) and Fu et al (2015) ^12^, ^14^ in the normal and OA patients‐derived knee cartilage tissues. The results showed that all but AC019109.1 were significantly increased (Figure 1C). For a better assessment of the role of lncRNA in IL‐1β‐induced OA in vitro, we isolated and cultured primary human chondrocytes from traumatic amputation of OA patient‐derived knee joints. In addition to the morphology, the chondrocyte phenotype of the primary cultures was confirmed by immunofluorescence of type II collagen and Aggrecan which are two major components of the extracellular matrix (ECM) of articular cartilage^19^ (Figure 1D). IL‐1β exhibited a dose‐dependent growth inhibitory effect on both the primary OA chondrocytes and chondrosarcoma cell line SW1353 (Figure 1E). As IL‐1β at 10 ng/mL concentration exerted a robust inhibition comparable to the higher one tested, it was adopted for the subsequent in vitro experiments. Upon treatment with IL‐1β, we detected that RP11‐364P22.2 was mostly upregulated in both cell types in comparison with the untreated cells (Figure 1F). RP11‐399K21.10 was also significantly induced in the primary chondrocytes but not SW1353 cells. Neither RP11‐432M8.8 nor AC019109.1 were responsive to IL‐1β exposure (Figure 1F). Therefore, we chose RP11‐364P22.2 as the target lncRNA for the following studies.

### 3.2 IL‐1β inhibits chondrocytes proliferation and induces apoptosis in vitro

**FIGURE 2:**
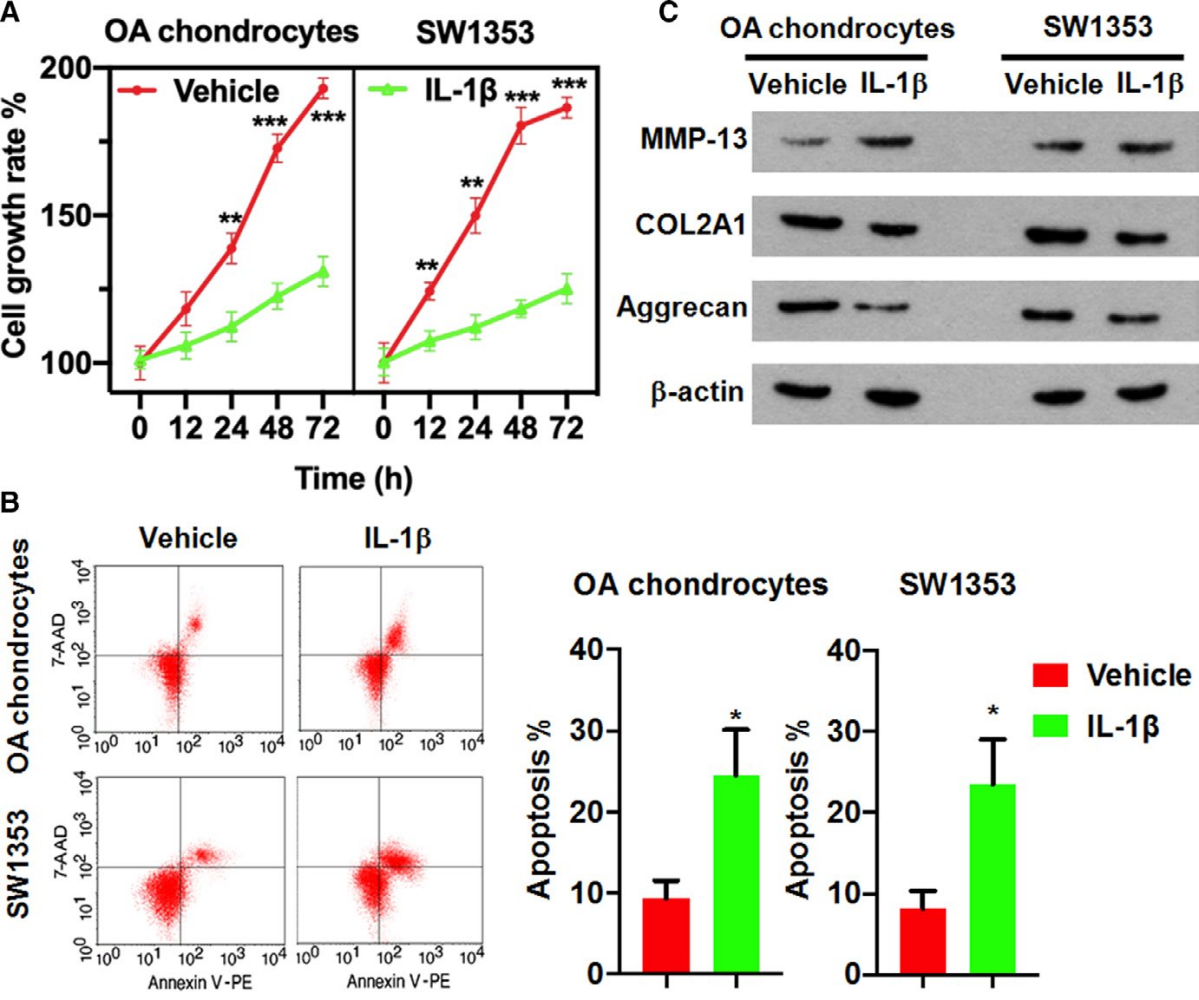
IL‐1β exhibited catabolic effects in chondrocytes. A, MTT assay showing growth retardation of chondrocytes in the presence of IL‐1β. B, Annexin V/7AAD apoptosis analysis showing the induced apoptosis in chondrocytes upon treatment with IL‐1β. C, Western blot assay showing that IL‐1β treatment blocked the synthesis of type II collage and Aggrecan but promoted the synthesis of collagenase 3 in chondrocytes

Growing evidence suggests that chondrocyte cell death occurs and contributes to OA development.^20^ For a better assessment of the role of lncRNA in IL‐1β‐induced OA, we firstly validated the catabolic effects of IL‐1β in our in vitro chondrocyte culture system. In line with many previous studies, both the IL‐1β‐treated primary OA chondrocyte and SW1353 cells exhibited a severe growth retardation (Figure 2A) and increased apoptosis (Figure 2B). IL‐1β treatment also blocked the synthesis of the key chondrocyte structural proteins of type II collage and Aggrecan but promoted the synthesis of collagenase 3 (MMP‐13) which elicits a destructive effect on cartilage components^21^ (Figure 2C). Taken together, these data demonstrated that the IL‐1β‐treated primary OA chondrocyte and SW1353 chondrosarcoma cells served as a robust in vitro model mimicking the degenerative effect of osteoarthritic cartilage.

### 3.3 RP11‐364P22.2 is essential for the effects of IL‐1β in chondrocytes

**FIGURE 3:**
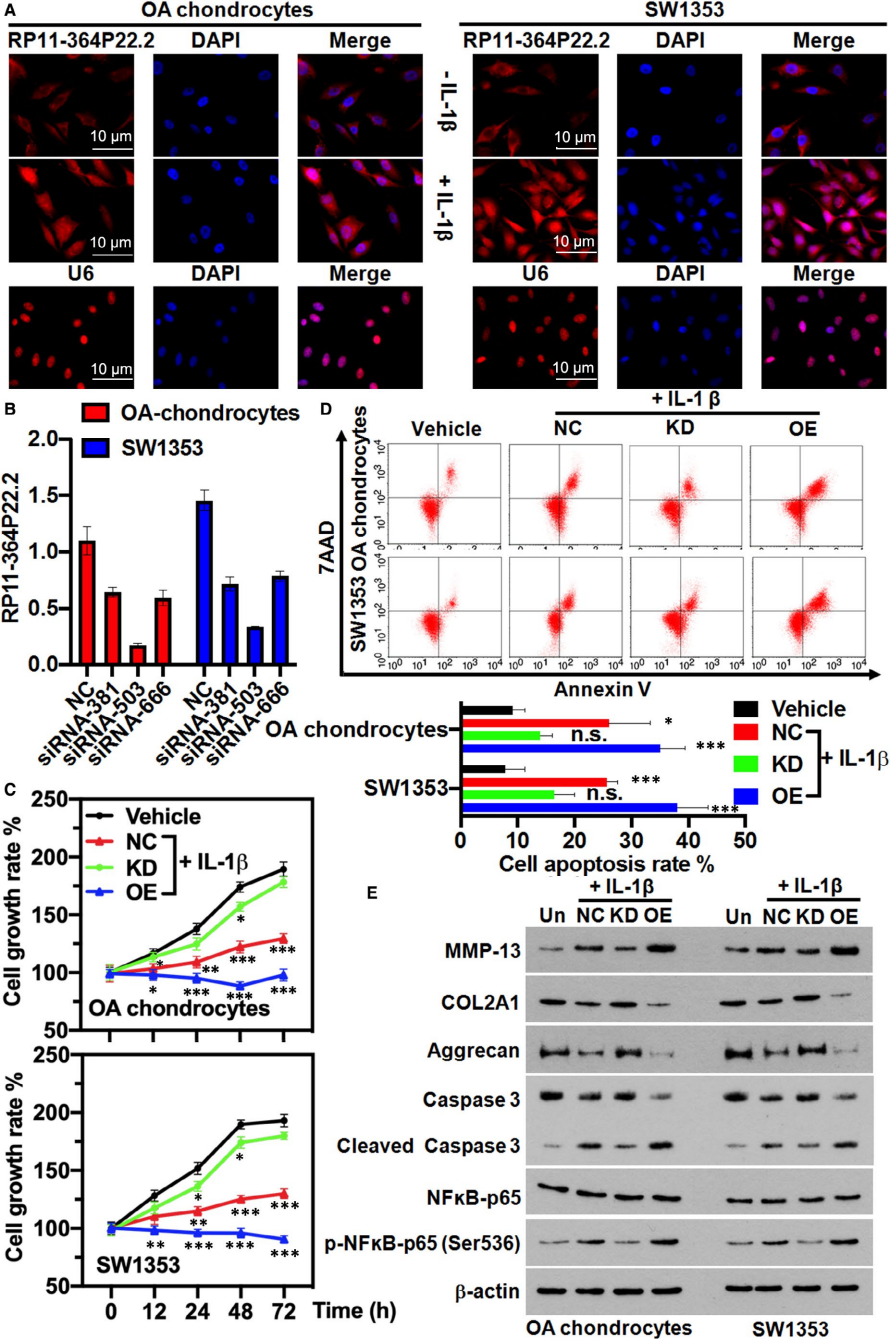
The catabolic effects of IL‐1β in chondrocytes requires RP11‐364P22.2. A, RNA FISH results showing the cellular localization of RP11‐364P22.2 in the chondrocytes before and after IL‐1β treatment. U6 was used as a control for nuclear localization. B, qPCR validation of RP11‐364P22.2 knock‐down efficiency in chondrocytes by different siRNAs. C, Time‐dependent cell proliferation determined by MTT assay. The growth inhibitory effect of IL‐1β in chondrocytes was ameliorated with depletion but aggravated upon overexpression of RP11‐364P22.2. KD, knock‐down; NC, negative control; OE, overexpression. D, Representative flow cytometry plots showing knock‐down of RP11‐364P22.2 abolished while its ectopic expression promoted IL‐1β‐induced apoptosis in chondrocytes. E, Western blot showing RP11‐364P22.2 depletion weakened while its overexpression enhanced the effects of IL‐1β in synthesis of the extracellular matrix proteins and collagenase 3 as well as activation of NF‐κB in chondrocytes. KD, knock‐down; NC, negative control; OE, overexpression; Un, untreated control

RP11‐364P22.2 was significantly upregulated under the stimulus of IL‐1β (Figure 1F). FISH results further demonstrated that RP11‐364P22.2 was only expressed at low abundance in the cytosol of the untreated chondrocytes, while the increased RP11‐364P22.2 was accumulated in both cytosol and nucleus of the IL‐1β‐treated chondrocytes (Figure 3A). Next, we optimized the conditions for siRNA‐mediated lncRNA knock‐down and applied the one with the highest knock‐down efficiency for the following loss‐of‐function studies (Figure 3B). Strikingly, the growth inhibitory and apoptotic effects of IL‐1β on the chondrocytes were abolished upon depletion of RP11‐364P22.2 (Figure 3C and D). On the contrary, ectopic expression of the lncRNA enhanced the functions of IL‐1β (Figure 3C and D). Loss of RP11‐364P22.2 also prevented IL‐1β from blocking synthesis of the structural proteins of type II collagen and Aggrecan as well as producing excessive MMP‐13, which were reversed when overexpressing the lncRNA (Figure 3E). The nuclear factor kappaB (NF‐κB) is a general and key inflammatory mediator in many cell types.^22^ The activation of NF‐κB‐dependent signalling pathway in the IL‐1β‐treated chondrocytes (Figure 3E) indicated that IL‐1β might intensify the inflammatory reaction during OA development. Interestingly, such role of IL‐1β relied to a large extent on the expression of RP11‐364P22.2 as knock‐down of the lncRNA attenuated while its forced expression promoted the activation of NF‐κB‐dependent signalling pathway (Figure 3E).

### 3.4 ATF3 is a target of RP11‐364P22.2

**FIGURE 4:**
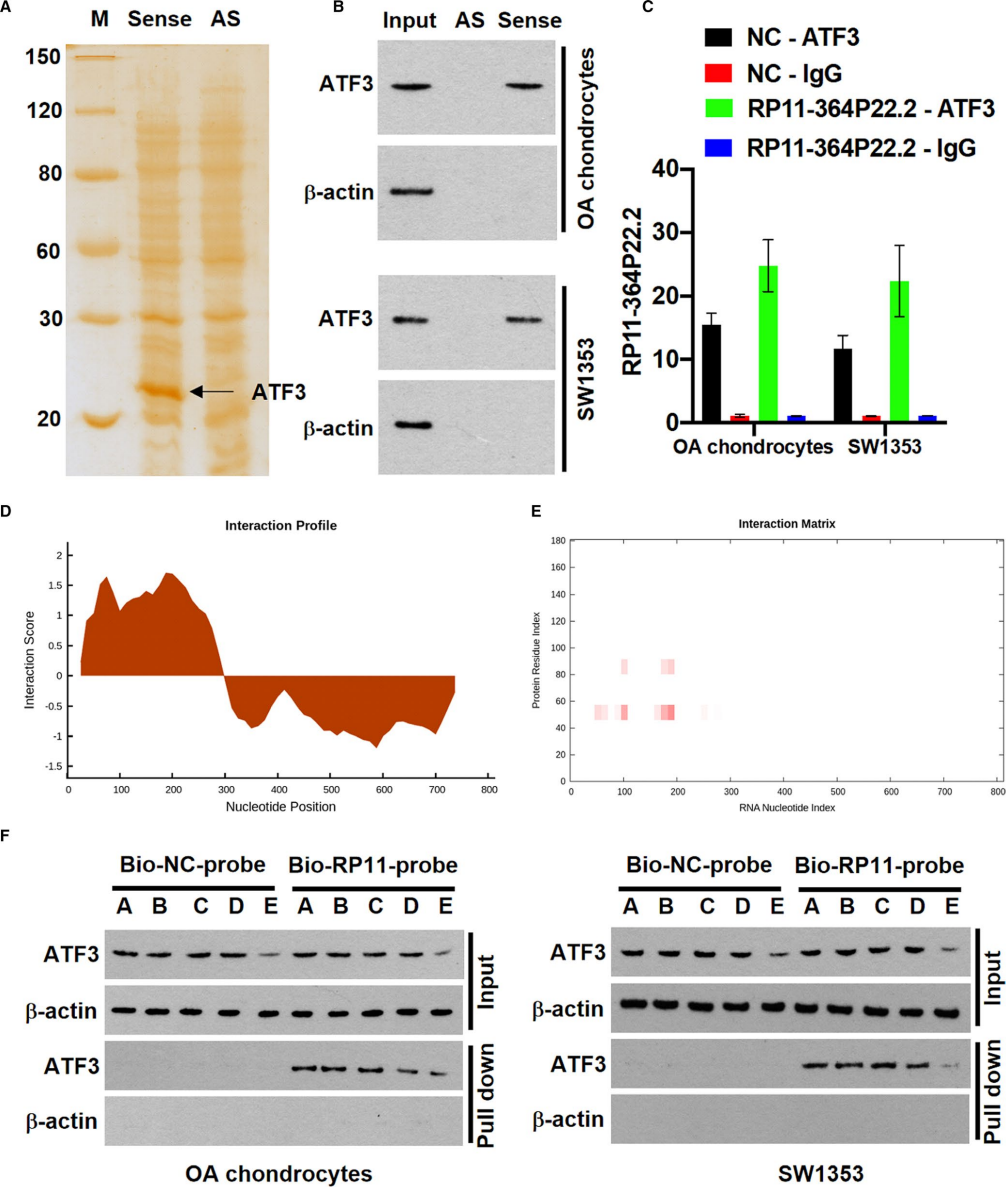
RP11‐364P22.2 physically binds to ATF3. A, Silver staining of the proteins pulled down from RP11‐364P22.2 sense and antisense RNAs incubated chondrocytes lysates. AS, antisense; M, protein marker. B, Western blot confirming the interaction between RP11‐364P22.2 and ATF3 in chondrocytes. C, qPCR determining enrichment of RP11‐364P22.2 in ATF3‐immunoprecipitated RNAs in control and RP11‐364P22.2 overexpressing cell lysate. Antibody against normal human IgG was included as a RIP negative control. D and E, interaction profile (D) and interaction matrix (E) demonstrating catRAPID fragments‐based prediction of interaction between RP11‐364P22.2 and ATF3. F, Western blot showing that transfection of the antisense oligo targeting 162‐227 nt of the lncRNA drastically decreased yield of the pull‐down ATF3. A, IL‐1β only; B‐E, chondrocytes were transfected with negative control oligo, antisense oligo targeting 37‐87 bp, 88‐138 bp and 162‐227 bp, respectively, followed by treatment with IL‐1β

The abundant cytosolic RP11‐364P22.2 in the IL‐1β‐treated chondrocytes reminds the protein modulator‐related function.^23^ Next, we performed RNA pull‐down coupled with mass spectrometry to identify the RP11‐364P22.2‐interacting proteins. Mass spectrum analysis of the unique protein bands appeared in the sense but not antisense RP11‐364P22.2‐incubated cell lysates by silver staining revealed ATF3 as a potential target (Figure 4A). Consistently, Western blot assay detected ATF3 in sense but not antisense RP11‐364P22.2 pull‐down cell lysates as well (Figure 4B). In turn, RIP assay using ATF3 antibody combined with qPCR analysis showed that ATF3 specifically precipitated RP11‐364P22.2 in chondrocytes cell lysates, and the precipitation was increased accordingly with an ectopic expression of RP11‐364P22.2 (Figure 4C).

We further performed an in silico catRAPID analysis ^24^ to define the domains in RP11‐364P22.2 that physically interact with ATF3. As the output interaction profile (Figure 4D) and interaction matrix (Figure 4E) files shown, three RNA regions including 37‐87 nucleotides (nt), 88‐138 nt and 162‐227 nt were highly scored for binding with the protein domain ranging from 31‐82 amino acids (aa) in ATF3. To test the predicted binding sites, we transfected the IL‐1β‐treated chondrocytes with antisense oligo targeting each fragment, respectively. The cell lysates were subject to pull‐down assay using Bio‐RP11‐364P22.2 probe. Western blot analysis showed that transfection of the antisense oligo targeting 162‐227 nt of the lncRNA largely decreased the yield of pull‐down ATF3 (Figure 4F).

### 3.5 RP11‐364P22.2 is required for protein stability and IL‐1β‐induced nuclear translocation of ATF3

**FIGURE 5:**
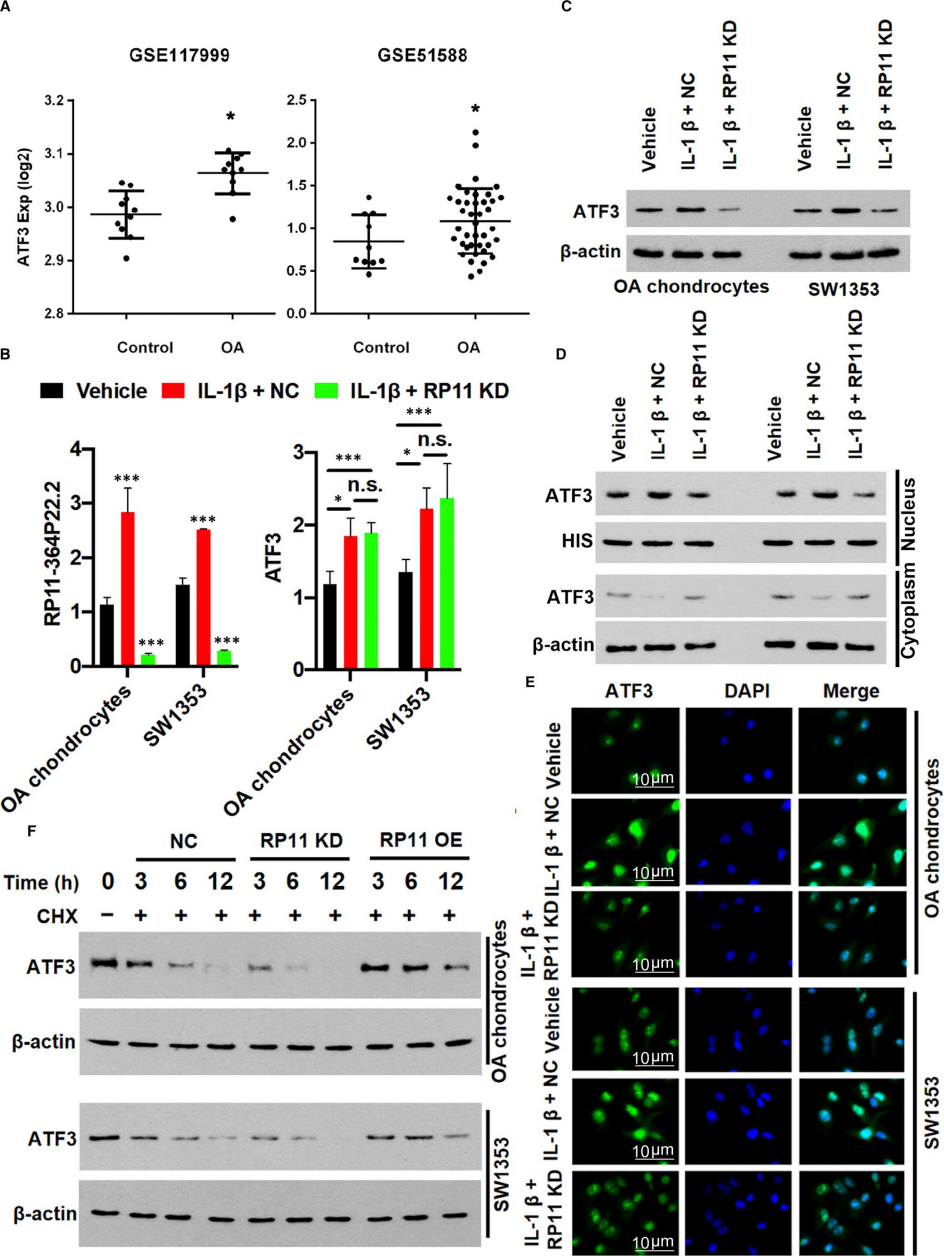
RP11‐364P22.2 facilitates IL‐1β‐induced ATF3 protein expression and nucleus translocation. A, Comparison of ATF3 expression in normal (Control) and OA cartilage tissues. B, qPCR validation of overexpression and knock‐down of RP11‐364P22.2 in chondrocytes (left), neither of which affected transcription of ATF3 (right). C, Western blot showing depletion of RP11‐364P22.2 decreased IL‐1β‐induced ATF3 protein expression. D and E, Western blot (D) and immunofluorescence (E) showing IL‐1β‐induced nucleus translocation of ATF3 in chondrocytes was abolished upon depletion of RP11‐364P22.2. F, Western blot showing depletion of RP11‐364P22.2 decreased while ectopic expression of the lncRNA increased the stability of ATF3 protein in chondrocytes. RP11, RP11‐364P22.2; KD, knock‐down; OE, overexpression

Previous studies had indicated the involvement of ATF3 in pathogenesis of early OA development.^25^, ^26^ Indeed, ATF3 expression was significantly increased in the OA cartilage tissues than non‐OA controls (Figure 5A). We continued to exam the effects of RP11‐364P22.2 in ATF3 expression. The qPCR results showed that while IL‐1β greatly induced ATF3 in the chondrocytes, depletion of RP11‐364P22.2 did not affect its expression at the transcription level (Figure 5B). Instead, induction of ATF3 protein by IL‐1β in the chondrocytes was largely abolished upon knocking down of RP11‐364P22.2 (Figure 5C). In chondrocytes, most majority of ATF3 was preferentially expressed in the nucleus with a small portion being detected in the cytoplasm (Figure 5D and E). IL‐1β treatment promoted a nuclear translocation of the cytosol ATF3 protein (Figure 5D and E), which was completely prohibited in the absence of RP11‐364P22.2 (Figure 5D and E). This was due largely to the fact that loss of RP11‐364P22.2 decreased ATF3 protein stability as indicated by the accelerated protein degradation in the presence of cycloheximide, a protein synthesis inhibitor in eukaryotes that inhibits translation elongation,^27^ while the opposite was true as well (Figure 5F).

### 3.6 RP11‐364P22.2‐stabilized ATF3 is required for the catabolic effects of IL‐1β in chondrocytes in vitro

**FIGURE 6:**
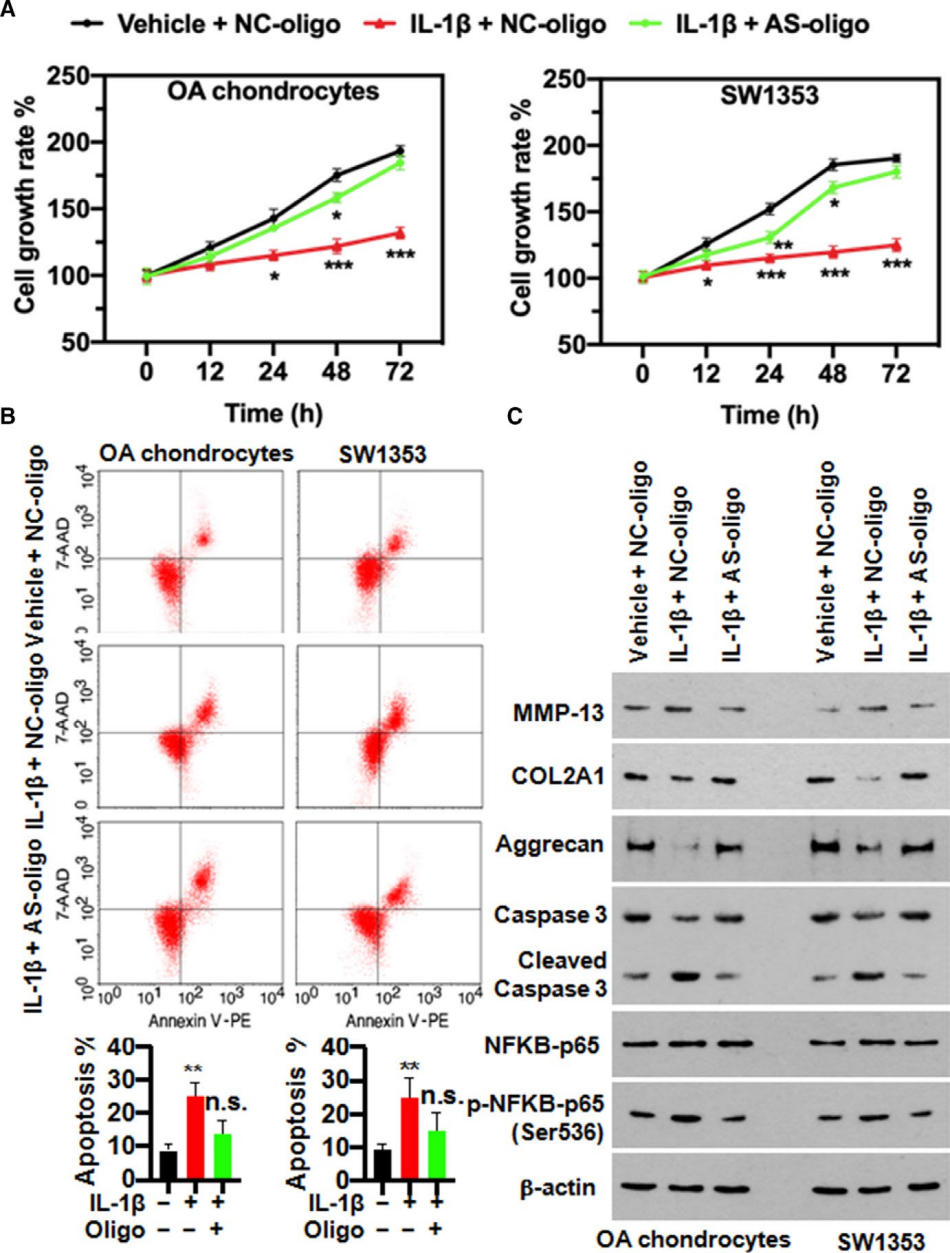
Physically binding of RP11‐364P22.2 to ATF3 is necessary for the catabolic effects of IL‐1β in chondrocytes. A, Time‐dependent MTT assay showing blocking the interaction between RP11‐364P22.2 and ATF3 by antisense (AS) oligo abolished IL‐1β‐caused chondrocytes growth retardation. B, Representative flow cytometry plots showing addition of antisense oligo abolished IL‐1β‐induced apoptosis in chondrocytes. C, Western blot showing the attenuated effects of IL‐1β in synthesis of the extracellular matrix proteins and collagenase 3 as well as activation of NF‐κB in chondrocytes upon blocking RP11‐364P22.2/ATF3 binding by antisense oligo. AS, antisense; NC, negative control

Next, we assessed the role of interaction between RP11‐364P22.2 and ATF3 in the IL‐1β‐induced effects in chondrocytes. By blocking the interaction with the antisense oligo targeting 162‐227 nt of the lncRNA, IL‐1β became incompetent in inhibiting proliferation (Figure 6A) and inducing apoptosis (Figure 6B) in the chondrocytes, which was verified by the less cleaved Caspase three after the treatment (Figure 6C). Meanwhile, it also prohibited degradation of the cartilage structural proteins of type II collagen and Aggrecan as well as synthesis of MMP‐13 in the chondrocytes (Figure 6C). Importantly, the IL‐1β‐activated NF‐κB signalling pathway was also reversed to a normal level upon breaking up the interaction between RP11‐364P22.2 and ATF3 (Figure 6C).

### 3.7 ATF3 is indispensable for the surgical DMM‐induced OA development in vivo

**FIGURE 7:**
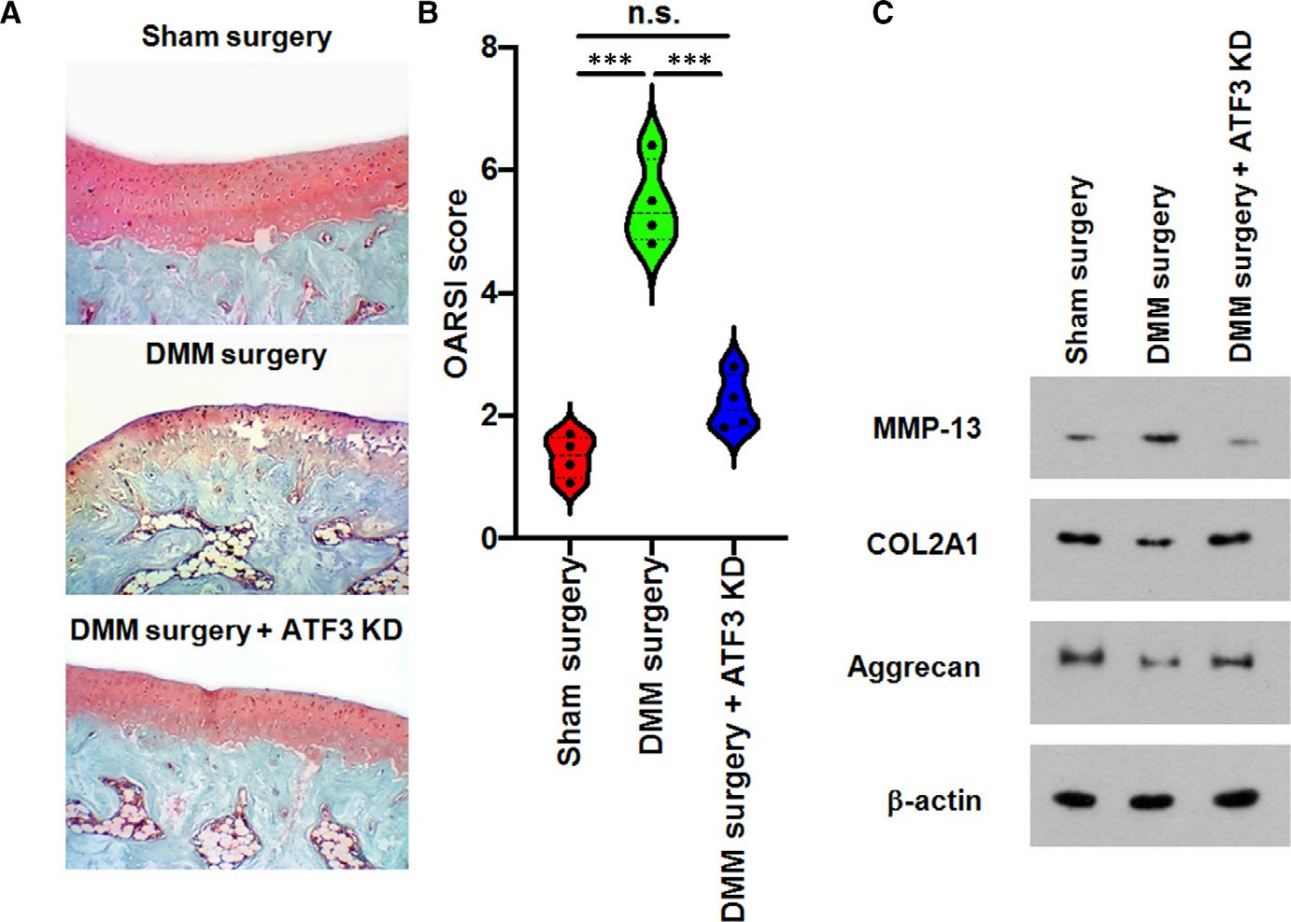
Depletion of ATF3 rescues surgical DMM‐caused OA development in vivo. A, Safranin‐O staining showing the severely developed OA symptoms in rats underwent surgical DMM was largely ameliorated by in vivo knock‐down of ATF3 in cartilage tissue of knee joints. KD, knock‐down. B, OARSI articular cartilage pathological scoring. KD, knock‐down. C, Western blot showing the increased synthesis of MMP‐13 and decreased expression of type II collage and Aggrecan in the cartilage tissues of rats underwent surgical DMM, which were restored by knocking down of ATF3

Our data from the in vitro chondrocytes‐based studies demonstrated that one of the key roles of RP11‐364P22.2 in IL‐1β‐induced OA was maintaining a high expression of ATF3 by stabilizing the protein. Next, we assessed the effect of ATF3 in OA development using an in vivo DMM rat model. As expected, rats subjected to the surgical DMM developed a typical OA by 8 weeks post‐surgery as indicated by the appearance of a series of pathological features including the rough and deformed cartilage articular surface with the disordered cell arrangement, incomplete or fuzzy tide line, partial fibrillation at articular cartilage as well as the loss of Safranin‐O staining on the medial tibial plateau (Figure 7A), which was graded a significantly higher Osteoarthritis Research Society International (OARSI) score compared to the sham group rats (Figure 7B). Western blot results also showed the increased synthesis of MMP‐13 and decreased expression of type II collage and Aggrecan in the cartilage tissues of these rats (Figure 7C). Strikingly, lentiviral shRNA‐mediated stable knock‐down of ATF3 in the cartilage tissue of knee joints retained in a great degree the Safranin‐O staining (Figure 7A) and synthesis of the chondrocyte structural proteins (Figure 7C) as indicative of an alleviated OA development after the surgical DMM. In accordance, cartilage surface in the ATF3‐depleted rats after the surgery was displayed with the regularly arranged cells and clear tide line but little fibrillation (Figure 7A). The OARSI score was significantly lower than that of in DMM group as well (Figure 7B). Taken all together, our study demonstrated that the highly expressed RP11‐364P22.2 is essential for the IL‐1β‐induced catabolic effects in cartilage tissue and chondrocytes by stabilizing ATF3.

## 4 DISCUSSION

Inflammation is an important concomitant throughout initiation and development of OA. Aetiologies such as joint injury, obesity and ageing that cause OA also contribute to inflammatory processes.^28^, ^29^, ^30^, ^31^ Emerging evidence has implicated the involvement of the pre‐inflammatory cytokine IL‐1β in the pathogenesis of OA. For example, expression of both IL‐1β and its converting enzyme was increased at the superficial and supper intermediate layers of articular cartilage, where histological degeneration occurs in human OA cartilage.^32^ Meanwhile, clinical observation found that the proportion of the IL‐1β to the natural IL‐1 receptor antagonist IL‐1Ra is positively associated with the grades of OA in patients.^33^ Therefore, our current study aimed to uncover key lncRNAs that mediate the detrimental effects of IL‐1β on chondrocytes function and integrity of ECM.

Chondrocytes are one of the major cellular targets of IL‐1β for their expression of IL‐1 receptor.^34^ Notably, it has detected abundant IL‐1 type I receptor in chondrocytes of patients with OA, which further enhances catabolic effects of IL‐1β by initiating diverse signalling pathways including Ca^2+^ surge, activation of NF‐κB as well as nuclear translocation of ATF and AP1, *etc* ^35^ Therefore, chondrocyte cell line provides a valuable in vitro model for investigating the molecular mechanism of IL‐1β function. SW1353 is a human chondrosarcoma cell line that has been widely employed for OA‐related functional studies. However, previous IL‐1β‐induced gene expression analysis suggested that SW1353 cells possess only limited potential in mimicking primary human adult articular chondrocytes.^36^ Indeed, we also observed a difference between the primary chondrocytes and this cell line when screening IL‐1β‐responsive lncRNAs (Figure 1E). Therefore, we established additional in vitro model using the primary human chondrocytes from articular cartilage of OA patients and conducted the paired assays in both primary and chondrosarcoma cells so as to ensure the accuracy of analyses.

Consistent with previous report that inflammatory cytokines induce ATF3 expression through NF‐κB pathway,^25^ we detected both the activated NF‐κB signalling pathway and upregulated ATF3 in the IL‐1β‐treated chondrocytes. Interestingly, RP11‐364P22.2 turned out to be essential for IL‐1β‐induced activation of NF‐κB pathway. We speculate that it might because of the post‐translational regulation of the lncRNA on ATF3. The amount of ATF3 protein dramatically decreased in the chondrocytes lack of RP11‐364P22.2, which in turn attenuates the phosphorylation of IκB and p65.^25^ Of note, it is ATF3 protein stability but not its transcription relies on RP11‐364P22.2 in chondrocytes, which is also indicated by the predicted binding site of RP11‐364P22.2 in ATF3. The output interaction matrix file of catRAPID analysis showed that the lncRNA binds to the domain ranging from 31‐82 aa of ATF3, which is known not as an active transcription binding domain. The decrease in ATF3 stability leads to a lower level of ATF3 protein due to the accelerated protein degradation. This might further impair degradation of IκBα which inhibits NF‐κB activation as it was reported that Kdo2‐Lipid A induced IκBα degradation was impaired in ATF3‐deficient MEF cells.^37^ Interestingly, the authors also found IκBζ which is an inducible nuclear protein that can selectively inhibit certain NF‐κB dimers was significantly upregulated in ATF3‐deficient cells. This might further contribute to the inactivation of NF‐κB as the activity of NF‐κB is controlled in a negative feedback loop by IκBζ.^38^

ATF3 enters the nucleus via nuclear localization signals (NLS) in the basic region that binds DNA.^39^, ^40^ Although ATF3 nuclear translocation occurs under many stimuli, the precise mechanism remains unclear. Interestingly, Takii et al showed that overexpressing ATF3 alone was not sufficient for its binding to IL‐6 promoter.^41^ Instead, ectopic ATF3 expression under a heat shock condition enhanced the binding by accelerating its nuclear translocation, indicating the translocation from the cytoplasm to nucleus could be mediated by certain chaperons or molecules. As discussed above, RP11‐364P22.2 binds to the transcription activity domain but not the basic region where NLS appears in ATF3. Therefore, it is tempting to investigate whether loss of the lncRNA affects the expression of chaperons mediating the translocation. In view of the studies by Takii et al, we think that although ATF3 has been detectable in the nucleus without IL‐1β, it might be inactive or incompetent in binding to the promoters of target genes. IL‐1β treatment not only increases mRNA and protein abundance of ATF3 in the chondrocytes, more importantly, it further promotes nuclear translocation and protein stability of ATF3. This could be one of the key events leading to the activation of ATF3. It was reported that ATF family of transcription factors all bind to DNA as homo‐ or heterodimers so as to increase stability of the proteins,^39^ highlighting the importance of protein stability in ATF3‐induced gene transcription. In the normal chondrocytes, ATF3 is subject to the proteasomal degradation and thus has relatively short half‐life. This is due partially to the low level of RP11‐364P22.2 in the normal chondrocytes. IL‐1β treatment de‐represses expression of the lncRNA, which stabilizes ATF3 protein and extended its exposure time to the nuclear gene promoters.

DMM is one of the most prevailing surgical injury models for in vivo pathogenesis of OA. Inflammation‐associated genes were found upregulated from 2 to 16 weeks post‐surgery in DMM rat.^42^ Interestingly, Glasson et al ^43^ observed an anesis of DMM‐caused OA in rats at 4 and 8 weeks post‐surgery by knocking out IL‐1β, which highlights the importance of IL‐1β in DMM‐induced OA. Unfortunately, because RP11‐364P22 orthologous genes were not found in rat, we knocked down ATF3 that has been proved to be a direct target of the lncRNA in current study to suggest the importance of RP11‐364P22.2/ATF3 regulatory axis in IL‐1β‐induced OA in human.

In conclusion, we identified in the current study that RP11‐364P22.2 is upregulated in OA cartilage tissue and chondrocytes and highly responsive to IL‐1β stimulus. The lncRNA participates in IL‐1β‐induced OA development and progression by stabilizing the key transcription factor ATF3 protein and promotes its nuclear translocation, which intensifies inflammatory reaction by activating NF‐κB signalling pathway and facilitates transcription of MMP‐13 to accelerate degradation of the ECM in cartilage tissue. Our data shed light on targeting RP11‐364P22.2/ATF3 axis for OA treatment.

## CONFLICTS OF INTEREST

The authors declare that they have no conflict of interest.

## AUTHOR CONTRIBUTIONS

**Xilei Li:** Data curation (equal); Funding acquisition (equal); Investigation (equal); Methodology (equal); Project administration (equal). **Yusheng Li:** Conceptualization (equal); Data curation (equal); Resources (equal); Software (equal). **Xucheng Yang:** Conceptualization (equal); Data curation (equal); Formal analysis (equal); Funding acquisition (equal); Investigation (equal); Methodology (equal); Project administration (equal). **Runzhi Liao:** Methodology (equal); Project administration (equal); Resources (equal); Software (equal); Supervision (equal). **Liang Chen:** Conceptualization (equal); Investigation (equal); Supervision (equal); Validation (equal); Visualization (equal). **Qulian Guo:** Project administration (equal); Resources (equal); Software (equal); Supervision (equal); Validation (equal); Visualization (equal). **Junxiao Yang:** Data curation (equal); Formal analysis (equal); Resources (equal); Software (equal); Writing‐original draft (equal); Writing‐review & editing (equal).

## Data Availability

The data sets generated/analysed in the present study are available upon reasonable request from the corresponding author.
